# Neutral sphingomyelinase (SMPD3) deficiency disrupts the Golgi secretory pathway and causes growth inhibition

**DOI:** 10.1038/cddis.2016.385

**Published:** 2016-11-24

**Authors:** Wilhelm Stoffel, Ina Hammels, Bitta Jenke, Erika Binczek, Inga Schmidt-Soltau, Susanne Brodesser, Astrid Schauss, Julia Etich, Juliane Heilig, Frank Zaucke

**Affiliations:** 1 Center of Molecular Medicine (CMMC), Laboratory of Molecular Neurosciences, Center for Biochemistry, Faculty of Medicine, University of Cologne, Cologne, Germany; 2 Cluster of Excellence, Cellular Stress Response in Aging-Related Diseases (CECAD), University of Cologne, Cologne, Germany; 3 Department of Pediatrics and Adolescent Medicine, Experimental Neonatology, Center for Biochemistry, Faculty of Medicine, University of Cologne, Cologne, Germany; 4 Dr Rolf M Schwiete Research Unit for Osteoarthritis, Orthopedic University Hospital, Friedrichsheim gGmbh, Frankfurt/Main, Germany

## Abstract

Systemic loss of neutral sphingomyelinase (SMPD3) in mice leads to a novel form of systemic, juvenile hypoplasia (dwarfism). SMPD3 deficiency in mainly two growth regulating cell types contributes to the phenotype, in chondrocytes of skeletal growth zones to skeletal malformation and chondrodysplasia, and in hypothalamic neurosecretory neurons to systemic hypothalamus–pituitary–somatotropic hypoplasia. The unbiased *smpd3−/−* mouse mutant and derived *smpd3−/−* primary chondrocytes were instrumental in defining the enigmatic role underlying the systemic and cell autonomous role of SMPD3 in the Golgi compartment. Here we describe the unprecedented role of SMPD3. SMPD3 deficiency disrupts homeostasis of sphingomyelin (SM), ceramide (Cer) and diacylglycerol (DAG) in the Golgi SMPD3-SMS1 (SM-synthase1) cycle. Cer and DAG, two fusogenic intermediates, modify the membrane lipid bilayer for the initiation of vesicle formation and transport. Dysproteostasis, unfolded protein response, endoplasmic reticulum stress and apoptosis perturb the Golgi secretory pathway in the *smpd3−/−* mouse. Secretion of extracellular matrix proteins is arrested in chondrocytes and causes skeletal malformation and chondrodysplasia. Similarly, retarded secretion of proteo-hormones in hypothalamic neurosecretory neurons leads to hypothalamus induced combined pituitary hormone deficiency. SMPD3 in the regulation of the protein vesicular secretory pathway may become a diagnostic target in the etiology of unknown forms of juvenile growth and developmental inhibition.

Phospholipids (PLs), sphingolipids (SLs) and cholesterol form the complex architecture of mammalian membrane lipid bilayers. In addition, PLs and SLs are substrates of membrane-associated phospholipases and phosphodiesterases, the reaction products of which act as lipid second messengers. Acid sphingomyelinase (aSMase, SMPD1) and neutral SMases (nSMases, sphingomyelin (SM) phosphodiesterases, SMPD2-5)^1, 2, 3, 4^ hydrolyze SM to phosphocholine and Cer. Cers are regarded lipid second messengers in rather divergent pathways of cellular signaling in growth and development, including triggering tumor-suppressive and anti-proliferative cellular processes.^5, 6^ However, this tenet has been challenged ^7, 8, 9, 10, 11, 12, 13^ and Cer functions have remained enigmatic.

The null-allelic *smpd1-* (Niemann-Pick, type A),^14, 15^ *smpd2-*^11^ and *smpd3-*^13^ mouse-mutants have served as unbiased genetic tools in studies on the systemic role of mammalian SMPDs in cellular SM metabolism and its pathophysiology. The current study elaborated the pivotal role of SMPD3 utilizing the *smpd3−/−* mouse, which is characterized by the retardation of systemic and skeletal growth and development. In wild-type mice, the *smpd3* mRNA is ubiquitously expressed.^2, 16^, The absence of SMPD3 in hypothalamic secretory neurons inhibited the secretion of proteo-hormones, slowed down the hypothalamus–pituitary–growth axis, and triggered systemic growth retardation resulting in a novel juvenile dwarf phenotype.^13^ The autonomous expression of SMPD3 in chondrocytes was shown by functional reconstitution of SMPD3 in *smpd3−/−* chondrocytes, expressing *smpd3* as transgene, driven by the chondrocyte-specific Col2a1 promoter in the *smpd3−/−* mutant.^16^

Here, we describe a novel molecular mechanism underlying the bifurcated systemic and cell autonomous SMPD3 deficiency. We first documented the dominant role of SMPD3 and defined the subcellular localization in the Golgi compartment (GC), imperative for unraveling the molecular role of SMPD3 in mammalian cells. Our finding is ad variance with the proposed plasma membrane (PLM) topology of SMPD3.^18, 19^

We next focused our study on the cell-specific growth regulation of SMPD3 in primary chondrocytes of skeletal growth plates of p16 control and *smpd3−/−* mice, corresponding to approximately age 4 years in human development.^20, 21^ Chondrocytes are competent secretory cells during the growth phase with an abundant secretion of extracellular matrix proteins (ECMs) for enchondral ossification in longitudinal growth.^20^ Chondrocytes in culture have proven most powerful in exploring molecular features of growth and development.^22^

We discovered the pivotal role of SMPD3 in Golgi vesicular protein transport. Inactivation of *smpd3* stalled Golgi protein transport, disrupted proteostasis, induced ER stress and compromised chondrocyte function, leading to apoptosis and ultimately to skeletal malformation and severe chondrodysplasia.

We determined the lipidome of primary chondrocytes of control and *smpd3−/−* mice in the growth phase as the structural platform for the functional analysis of SMPD3 in the Golgi secretory pathway (GSP). Our studies suggest a concerted action of SMPD3 and SMS1 in the Golgi complex, which maintains SM/phosphatidylcholine and ceramide (Cer) diacylglycerol (DAG) homeostasis during remodeling of the Golgi membrane lipid bilayer for vesicular transport. This homeostasis is perturbed in SMPD3-deficient GC.

Our studies delineate a novel function of SMPD3 in the lipid-driven formation of vesicle carriers in the GSP during growth and development, and provide insight into the molecular pathology of SMPD3 deficiency leading to an unprecedented mechanism of growth inhibition and to retarded development, manifested juvenile dwarfism and osteochondrodysplasia.

## Results

### SMPD3 is the key neutral sphingomyelinase, localized in detergent-insoluble membrane domains of the Golgi complex

**Figure 1:**
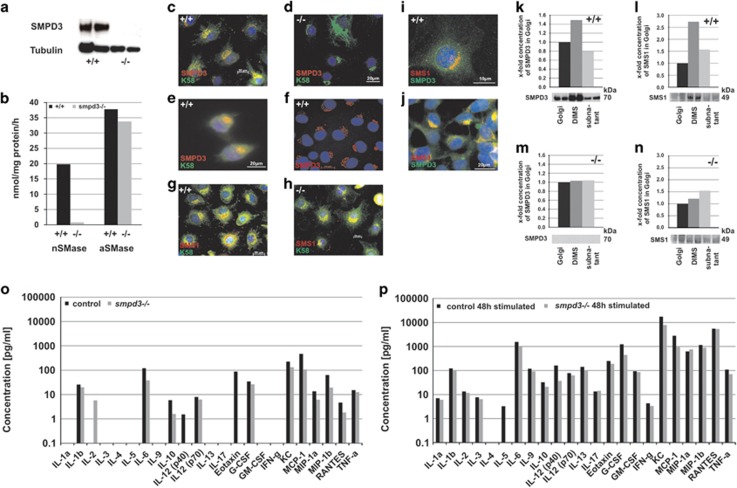
SMPD3 is the key nSMase segregated into DIMS of the GC. (**a**) Absence of SMPD3 in WB of lysates of *smpd3−/−* chondrocytes. (**b**) Quantitative assessment of nSMase and aSMase activity in control and *smpd3−/−* chondrocytes using radioactive enzyme assays, *N*=10. Fluorescence images demonstrate colocalization of SMPD3 (red) and K58 (green) (**c**) in control chondrocytes (**d**), absence of SMPD3 in *smpd3−/−* chondrocytes. (**e**) Colocalization of SMPD3 (red) and K58 (green) in peritoneal macrophages and (**f**) Golgi localization of SMPD3 in C57BL/6 embryonal fibroblasts, (**g** and **h**) colocalization of SMS1 (red) and K58 (green) in control and *smpd3−/−* chondrocytes, *N*=4. Colocalization of SMPD3 (green) and SMS1 (red) in (**i**) control chondrocytes and (**j**) smpd3 transfected HEK293 cells, *N*=3. (**k**-**n**) Representative densitometric evaluation of WB signals of SMPD3 and SMS1 in total Golgi-, DIM- and subnatant fractions of (**k** and **l**) control and (**m** and **n**) *smpd3−/−* p14 brain. In all, 100 *μ*g aliquots of total protein were applied to each lane. Signals were evaluated by densitometry and normalized to SMPD3 and SMS1 present in the Golgi fraction, *N*=4. The non-canonical secretory pathway was assayed within a multi-cytokine assay quantifying secretion of cytokines into the growth-medium of peritoneal macrophages, (**o**) unstimulated and (**p**) stimulated with LPS

We first ascertained the subcellular topology, a prerequisite for exploring the mechanistic role of SMPD3. Biochemical and immunohistochemical analyses conclusively proved the absence of SMPD3 in *smpd3−/−* chondrocytes (Figures 1a and d). SMPD3 topology was restricted to the Golgi complex in different control cell types: in primary control chondrocytes (Figure 1c), peritoneal macrophages (Figure 1e) and *C57Bl/6* EMFIs (Figure 1f). Colocalization with SMS1 and Golgi-specific marker K58 ascertained the allocation to the GC (Figures 1g and h). SMPD3 and SMS1 resided in the GC of control chondrocytes and SMPD3 overexpressing HEK293 cells (Figures 1i and j).

To further substantiate the Golgi topology of SMPD3 and of SMS1, we used mouse brain, which shows highest SMPD3 expression of all tissues.^2, 16^ The Golgi fraction of the premyelinating brain of (p14) control and *smpd3−/−* mice was further separated into detergent (Triton X-100)-insoluble membrane domains (DIMs) and subnatant for WB (Figures 1k and n). More than two-thirds of total SMPD3 resided in DIMS of control GC (Figure 1k), SMPD3 was absent in *smpd3−/−* GC (Figure 1m). Similarly, two-thirds of total SMS1 was concentrated in DIMs of control GC (Figure 1l), but was equally distributed in SMPD3-deficient DIMs and subnatant (Figure 1n).

To explore whether SMPD3 is required selectively for the canonical Golgi-mediated or non-canonical secretory pathway, we analyzed the secretion of several cytokines into the medium of control and *smpd3−/−* peritoneal macrophages, unstimulated (Figure 1o) and stimulated (Figure 1p) with lipopolysaccharide (LPS). The unchanged secretion of cytokines in SMPD3-deficient macrophages clearly indicated SMPD3 function to be restricted to the canonical GSP.

A valuable tool in dissecting Golgi localization, morphology and vesicle formation is exposure of chondrocytes to Brefeldin (BFA). We followed the time-resolved disintegration by IHC of the GC of SMPD3 and K58 (Supplementary Figure S2a), and Col2a and cartilage oligomeric matrix protein (COMP), in control and *smpd3−/−* chondrocytes (Supplementary Figure S2b). Golgi membrane stacks disintegrated, vesiculated and fused with ER membranes within 5–30 min.

We then assessed the contribution of bona fide SMPD4 (ref. 3) to cellular nSMase activity in HEK293 cell clones, stably transfected with full-length *smpd4-egfp*, with threefold to an eightfold overexpression, documented by qRT-PCR (Supplementary Figure S1a). SMPD4-EGFP colocalized largely with K58 in the GC (Supplementary Figure S1c). Surprisingly, the sensitive radioactive nSmase assay revealed an unchanged basal nSMase activity in the postnuclear fraction of all *smpd4-egfp* overexpressing cell clones (Supplementary Figure S1b). Our biochemical experiments and IHC were unable to substantiate nSMase activity of SMPD4.

### The SMPD3-SMS1 cycle regulates SM homeostasis and the Cer- and PLC-independent DAG pool in the GC

Current techniques preclude time and space resolution between the transfers of *de novo* synthesized Cer from the ER into the GC, and Cer, released locally by SMPD3 hydrolysis from SM of Golgi membrane domains. To get insight into the metabolic interrelationship of SM/Cer and PC/DAG, we focused on their analysis in control and *smpd3−/−* primary chondrocytes in culture. We next analyzed the PL-classes of control and *smpd3−/−* chondrocytes, which were separated by high-performance thin layer chromatography (HPTLC).

**Figure 2:**
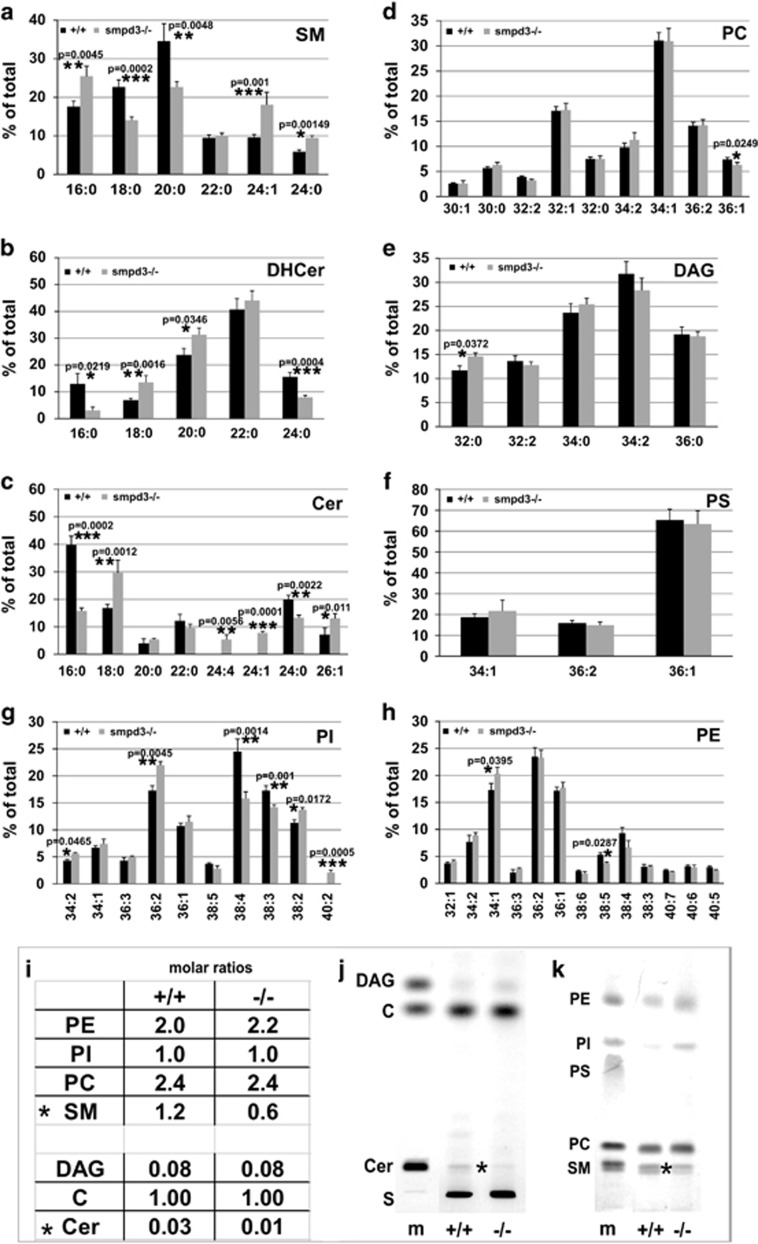
Molar ratios of SM and Cer are reduced in Golgi-lipidome of *smpd3−/−* chondrocytes. MS/MS profiling of (**a**-**h**) species of SM (**a**), dihydroceramide (DH-Cer) (**b**), Cer (**c**), PC (**d**), DAG (**e**), phosphatidylserine (PS) (**f**), PI (**g**) and phosphatidylethanolamine (PE) (**h**) of Golgi fraction of control and *smpd3−/−* chondrocytes. (**i**) Molar ratio of main PL classes (PC, PI and PE) and SM, Cer, C and DAG. (**j** and **k**) Thin layer chromatographic separation of lipid extract of control and *smpd3−/−* chondrocytes. Solvent system: cyclohexane/ethylacetate 3:2 v/v (**j**); two runs in chloroform/ethanol/water/triethylamine 30:35:7:35 v/v/v (**k**). *N*=3

Basically, Cer and DAG concentrations in the lipidome are very low. In *smpd3−/−* chondrocytes, the molar ratios of SM and Cer were reduced to one-half and one-third, respectively (Figures 2i and k).

Mass spectrometry, using selective ion monitoring (SIM), was applied for identification and quantification of HPTLC-separated Cer bands (m/z 264 and m/z 266), respectively. In GC, the pattern of fatty acid substituents of DH-Cer of control and *smpd3−/−* chondrocytes represents *de novo* synthesized DH-Cer, which is markedly different from that of Cer. DH-Cer in control and *smpd3−/−* chondrocytes contained only saturated 16:0–22:0 acyl-groups, Cer species predominantly very long chain 24:0, 24:4 and 26:1-acyl-residues as substituents (Figures 2b and c).

PC, the donor in the SMS1 catalyzed transfer of the phosphoryl-choline head group for reconstitution of SM, in Golgi of control and *smpd3 −/−* chondrocytes lacked polyunsaturated fatty acid substituted DAGs, which are closely similar to species in the pool of free DAGs. Sn1-18:0-sn2-20:4-DAG, released by PLC*γ*-specific hydrolysis of PLM-bound PIP2 (Supplementary Figure S3f) was hardly detectable in the DAG pool of the Golgi complex (Figure 2e). The hydrophobic DAG core of PS, PI and PE in the Golgi membrane bilayer of chondrocytes of p16 control and *smpd3−/−* mice remained unchanged (Figures 2f and h).

### SMPD3 deficiency disrupts the GSP

**Figure 3:**
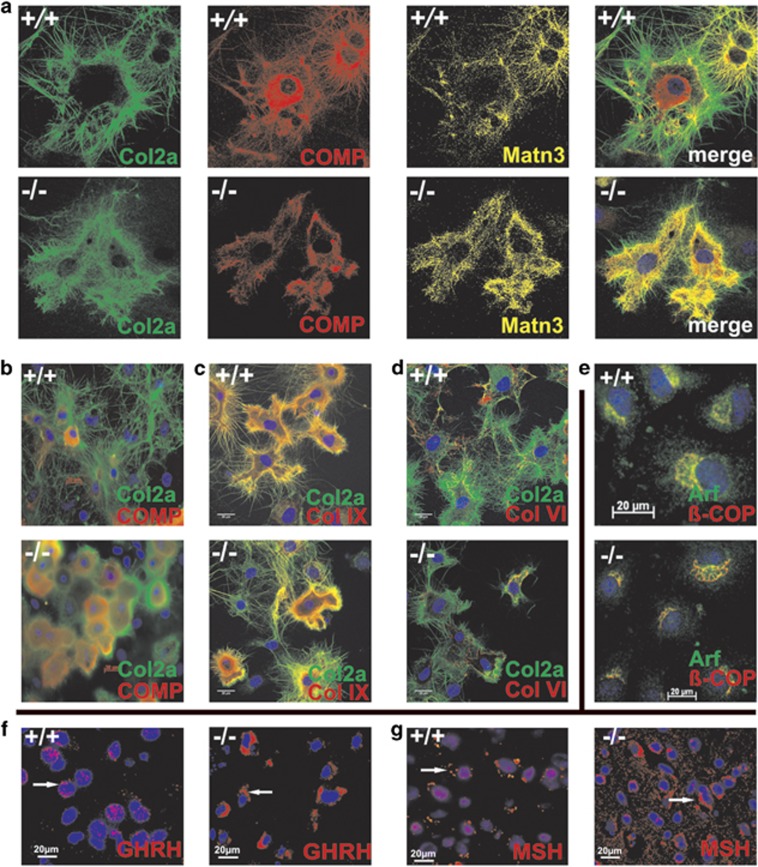
SMPD3-deficient primary chondrocytes display inhibited secretion of ECM proteins. Fluorescence images of p16 control and *smpd3−/−* primary chondrocytes, using following antibodies for triple staining: (**a**) Col2a (green), COMP (red), and Matn3 (yellow), and double staining: merged images of (**b**) Col2a (green), COMP (red), (**c**) Col2a (green), ColIX (red), (**d**) Col2a (green), ColVI (red), and (**e**) Arf (green), *β-*Cop1 (red) double stained primary chondrocytes in culture. Inhibited GSP in hypothalamic secretory neurons of control and *smpd3−/−* mice: fluorescence images of (**f**) GHRH secreting neurons in control, storage of GHRH in *smpd3−/−* neurons using anti-GHRH, (**g**) MSH in control and storage of MSH in proopiomelanocortin (POMC) expressing *smpd3−/−* neurons. *N*=3

Primary chondrocytes of control and *smpd3−/−* mice (p16) in culture show an abundant ECM protein synthesis, macro-vesicular transport and secretion. We followed Golgi vesicular transport and secretion of major ECM proteins, Col2a, the main fibrillar collagen species, perifibrillar COMP, Matrilin (Matn) 3, ColIX and ColVI in primary chondrocytes of p16 control and *smpd3−/−* mice by IHC (Figures 3a and h). Control chondrocytes effectively secreted Col2a, Matn3, ColVI and ColIX and formed a high-density ECM network. Col2a secretion was stalled in *smpd3−/−* chondrocytes and the intercellular fibrillar network nearly absent. COMP was distributed throughout the intracellular space of control chondrocytes. Arf and *β-*Cop1, markers of Golgi small vesicular transport carriers, displayed indistinguishable Golgi localization in control and *smpd3−/−* chondrocytes (Figure 3e).

IHC of the other dominant growth regulating cell type, hypothalamic neurosecretory neurons in *arcuate N.* and *periventricular N.,* using antibodies against growth hormone-releasing hormone (GHRH) and melanocyte-stimulating hormone (MSH), revealed *smpd3−/−* neurons heavily loaded with proteo-hormones, but low abundance in control neurons (Figures 3f and g), which confirms previous immunohistochemical results.^13^

### Inhibited protein transport, dysproteostasis, ER stress and apoptosis in SMPD3-deficient chondrocytes

We next studied the cellular response to the stalled protein transport along the GSP. ER stress is measured by activation of unfolded protein response (UPR) and visualized by the accumulation of misfolded proteins in the lumen of the tubular ER system directly by transmission electron microscopy (EM) (for review Oslowski and Urano^24, 25^ and Riggs *et al.*^26^).

**Figure 4:**
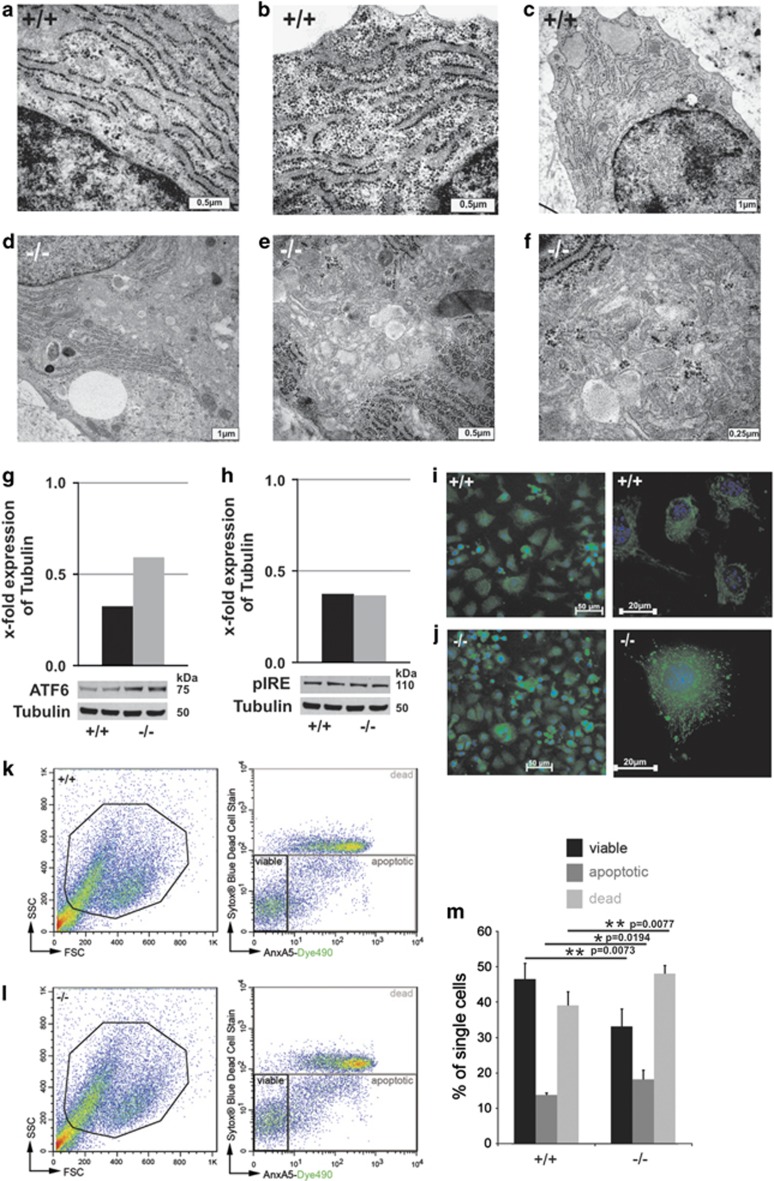

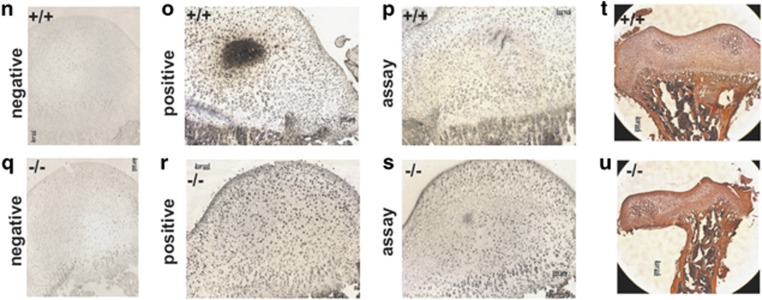
Perturbed proteostasis, UPR, ER stress and apoptosis in primary *smpd3−/−* chondrocytes. (**a**-**c**) EM displays regular rER in control chondrocytes and (**d**-**f**) dilated and giant ER cisternae in *smpd3−/−* chondrocytes, *N*=3. (**g** and **h**) WB of control and *smpd3−/−* chondrocytes of ATF6 and pIRE, *N*=3. (**i** and **j**) Annexin V staining of control and *smpd3−/−* chondrocytes. Flow cytometry of single-cell primary chondrocytes from control (upper panel) and *smpd3−/−* (lower panel) mice, using AnxA5-Dye490 and SYTOX Blue Dead Cell Stain. Representative dot plots are shown. (**k**) Cell debris were excluded according to size (FSC) and granularity (SSC), followed by gating of single cells (data not shown). (**l**) AnxA5^(-)^/Sytox Blue^(-)^ viable, AnxA5^(+)^/Sytox Blue^(-)^ apoptotic and AnxA5^(+)^/Sytox Blue^(+)^ dead cells were detected. (**m**) Mean percentage of AnxA5^(-)^/Sytox Blue^(-)^ viable, AnxA5^(+)^/Sytox Blue^(−)^ apoptotic and AnxA5^(+)^/Sytox Blue^(+)^ dead cells ±S.D. is given for control (+/+) and *smpd3−/−* animals, *N*=4. TUNEL assay in sections of radial epiphysis. (**n** and **q**) negative control, (**o** and **r**) positive control and (**p** and **s**) assay of control and *smpd3−/−* chondrocytes, *N*=4. (**t** and **u**) Light microscopy of HE–stained paraffin sections (5 *μ*m) of decalcified tibial epiphysis of control and *smpd3−/−* mice (4mo), *N*=4

EM of p16 control chondrocytes revealed normal rough endoplasmic reticulum (rER) tubular network (Figures 4a and c), but dilated and giant ER cisternae in s*mpd3−/−* chondrocytes, the cytoplasm filled with macro-vesicular structures, displacing and disrupting the rER (Figures 4d and f). Enhanced expression of ER stress sensor/transducer activating transcription factor 6 (ATF6), following stress-induced proteolysis indicated activation of UPR in *smpd3−/−* chondrocytes, but UPR responder protein pIRE remained unchanged in WB (Figures 4g and h). Annexin V staining of *smpd3−/−* chondrocytes (Figure 4j), FACS analysis of single-cell control and *smpd3−/−* p16 chondrocytes in culture (Figures 4k and m) and the terminal deoxynucleotidyl transferase dUTP nick end labeling (TUNEL) assay ^27^ in sections of long bone epiphyses of *smpd3−/−* mice (Figures 4n and s), monitored onset of apoptosis, which resulted in severe osteo-chondrodysplastic malformation, paradigmatically documented in sections of decalcified femural epiphysis of *smpd3−/−* mice (Figure 4u).

### *Smpd3−/−* gene expression in primary chondrocytes

**Figure 5:**
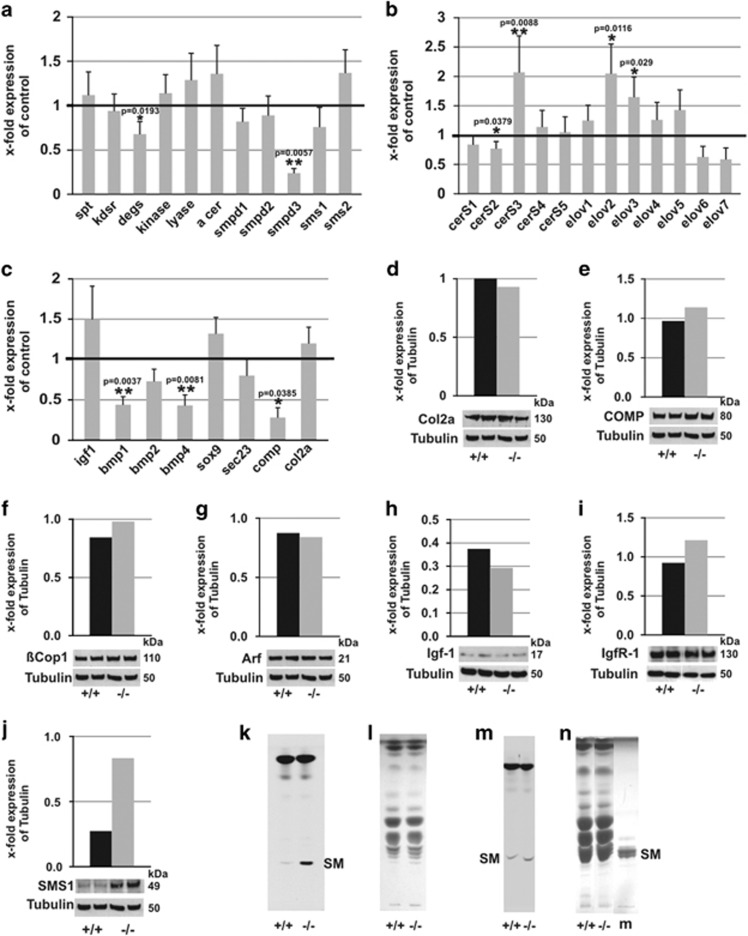
Gene expression profiling of enzymes of SL metabolism, transcription factors, growth factors and ECM proteins in control and *smpd3−/−* chondrocytes. Real-time PCR of control and *smpd3−/−* chondrocytes of (**a**) *spt2 (serine-palmitoyl-CoA transferase), kdsr (3-ketodihydrosphingosine reductase), degs (4t-dihydrosphingosine desaturase), kinase (sphingosine-1-kinase), lyase (sphingosine-1-phosphate lyase), a-cer (acid ceramidase), smpd1, smpd2, smpd3, sms1* and *sms2.* (**b**) *cerS 1-5* and *elovl1-7 (fatty acid elongases)* and (**c**) *igf1, bmp1, 2, 4, sox9, sec23, comp* and *col2a*. Primers are listed in Supplementary Table S1. Expression was normalized to HGPRT, relative expression calculated using the 2-ΔΔCt method,±S.D., *N*=5. (**d**-**j**) Densitometry of WB signals of Col2a, COMP, *β-*Cop1, Arf, Igf-1, IgfR-1 and SMS1 in chondrocyte lysates of control (black) and *smpd3−/−* mice (gray), *N*=3. SMS1 activity in post-mitochondrial fraction of control and *smpd3−/−* chondrocytes (**k**) and kidney (**m**) using NBD-fluorescence C_6_-Cer (6-((*N*-(7-nitrobenz-2-oxa-1,3-diazol-4-yl)amino)hexanoyl)sphingosine) as substrate. HPTLC-separation and visualization as inverted images. (**l** and **n**) Lipid classes in k and m were visualized by charring, *N*=3

Next, we studied steady-state gene expression in chondrocytes of p16 control and *smpd3−/−* littermates by real-time PCR of (a) key enzymes of SM metabolism (Figure 5a), of *ceramide synthases* (*cerS*) and *fatty acid elongases* (*elovl)* (Figure 5b), (b) growth and transcription factors regulating chondrocyte differentiation, (c) ECM proteins and (d) *sec23*, essential for protein transport (Figure 5c). Expression of *comp*, *bone morphogenetic protein 1 (bmp1)*, a pro-collagenase, and growth factor *bmp4*, required for cartilage formation was downregulated (Figure 5c).

Complementary WB analysis of structural proteins Col2a, COMP, Golgi-transport regulatory proteins *β*-Cop1 and Arf, Igf-1, IgfR-1 and SMS1 (Figures 5d and j). SMS1 expression was increased nearly threefold (Figure 5j), matching the increased SMS1 enzyme activity in the lysate of *smpd3−/−* chondrocytes (Figure 5k).

Equal amounts of lipid total extracts of enzyme assays were separated by HPTLC and visualized by charring lipid bands (Figure 5l), different from enzyme assay of kidney, which shows equal amounts of *de novo* synthesized fluorescent SM (Figures 5m and n).

## Discussion

This study is focused on the mechanism underlying SMPD3 deficiency, which causes systemic and cell-specific growth inhibition, a novel form of juvenile dwarfism. We used the unbiased *smpd3−/−* mouse model. In this study, primary chondrocytes in culture of control and *smpd3−/−* mice – as compelling *in vitro* system – were instrumental in unraveling the molecular mechanism underlying the crucial function of SMPD3 in the GSP. The absence of SMPD3 suppressed ECM protein transport and secretion, disrupted proteostasis, activated UPR and ER stress and finally apoptosis, which is reflected phenotypically in skeletal growth inhibition and joint malformation. Translation of this mechanism into disruption of the GSP in hypothalamic proteo-hormones secreting neurons provides a molecular interpretation of the previously described hypothalamus induced combined pituitary hormone deficiency, underlying the systemic hypoplasia of the *smpd3−/−* mutant.^13^

Exploring the function of SMPD3 in the non-canonical GSP, we quantified cytokine secretion in the medium of control and *smpd3−/−* peritoneal macrophages. The unaltered secretion indicated the restriction of SMPD3 function to the canonical GSP (Figures 1o and p).

SMPD3 is the dominant among the four mammalian nSMases (SMPD2-5),^1, 2, 3, 4^ contributing >90% of total cellular nSMase activity in all mouse tissues, followed by SMPD2.^13^ Gene expression, protein expression and nSMase enzyme activity studies on SMPD4 overexpressing HEK293 cells, reported here, preclude SMPD4 as nSMase (Supplementary Figure S1).

Conclusive data on the subcellular topology are a prerequisite for functional studies, as this issue has been controversially discussed and the PLM proposed as the scaffold of SMPD3.^18, 19^ The immune-histochemical and biochemical studies, reported here, further suggested the localization and association of SMPD3 and SMS1 in the Golgi complex.

SMPD3 and SMS1 are concentrated in DIMs of the GC of control chondrocytes. In the absence of SMPD3, SMS1 segregates from DIM domains, which disturbed structure of DIMs and deregulate SM synthesis in the GC (Figure 1n). SMS1 protein concentration and enzyme activity were increased in lysates of *smpd3−/−* chondrocytes (Figures 5k and l). SM and Cer concentrations were reduced (Figure 2i).

Free DAG species resembled that of the DAG core of PC, the donor substrate in the SMS1 reaction. SMPD3 deficiency caused no SM storage and the phospholipidome in all tissues of *smpd3−/−* mice remained unimpaired, unlike the fatal lysosomal SM storage in the *smpd1−/−* mouse,^14, 15^ a mimicry of human Niemann–Pick disease (Figures 2a and h).^14, 15^

### Disrupted protein transport in GSP of SMPD3-deficient chondrocytes

Chondrocytes are competent secretory cells, actively synthesizing and secreting ECM proteins for epiphyseal cartilage architecture^28^ during the growth phase. The impaired GSP in *smpd3−/−* chondrocytes causes cytoplasmic accumulation of dominant ECM proteins: fibrillar Col2a, perifibrillar COMP, Matn3, ColVI and ColIX (Figures 3a and d). Dysproteostasis activates UPR (Figure 4g), unable to alleviate ER stress, but induces premature apoptosis in *smpd3−/−* chondrocytes (Figures 4i and u).

EM convincingly unveiled the morphological changes, reflecting these processes: the dense coherent rER tubular system in control chondrocytes (Figures 4a and c) is contrasted by inflated cisternae of the rER network, loaded with polymorphic macro-vesicular, fibrillar and granular structures dispersed in the cytoplasm of *smpd3−/−* chondrocytes (Figures 4d and f). This finally leads to skeletal growth retardation, malformation and chondrodysplasia (Figures 4t and u).

Gene and protein expression of representative ECM proteins Col2a and COMP, Golgi-micro vesicular transport proteins Arf and *β-*Cop1 and of growth factor Igf1 and IgfR were inconspicuous (Figures 5d and i). Expression of growth factors, *bmp1, bmp4* and ECM protein *comp*, respectively, is downregulated (Figure 5c). Secreted BMP1 acts as Ca^2+^ and Zn^2+^-dependent metalloproteinase processing procollagen I for ECM organization. SMPD3 is selectively required for the canonical Golgi-mediated secretory pathway. Evidence for unimpaired secretion of non-canonical secretory proteins was provided by multi-cytokine assay, quantifying of 23 cytokines, secreted into the growth-medium of unstimulated and LPS-stimulated peritoneal macrophages of control and *smpd3−/−* mice, which revealed closely similar concentrations, presented in Figures 1o and p.

### SMPD3 transiently modifies the Golgi membrane for vesicular transport

The Golgi complex is a budding organelle with domain formation by lateral diffusion. Coatomer complex COPI and II drive budding, fission and fusion in the formation of small vesicular transport carriers.^29^ The function of the SMPD3-SMS1 cycle in the formation of large pleiomorphic carriers for intracellular transport and secretion of ECM proteins in the GSP of chondrocytes, reported here, is a novel facet in studies addressing this enigmatic process.

**Figure 6:**
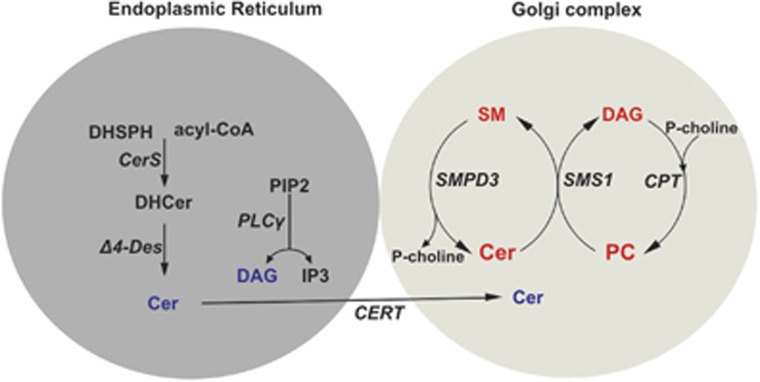
Proposed mechanism of the SMPD3-SMS1 cycle in the Golgi complex. Proposed mechanism of maintenance of homeostasis and regulation of SM, PC, Cer- and DAG-pools in the SMPD3-SMS1-cycle of the Golgi complex

The central role of SMPD3 and its concerted activity with SMS1 in the SMPD3-SMS1 cycle, which maintains homeostasis of SM metabolism in the GC, is delineated in Figure 6.

Current methodology precludes space- and time-resolved analysis of Golgi membrane domains undergoing remodeling during Golgi vesicular transport. Backed by biochemical, cellular and morphological finding, we conclude that the SMPD3-SMS1 cycle generates a PLC-independent DAG pool in the GC. It is of note that the species of this DAG pool largely reflect those of the major PC species, donor substrates in the SMS1 catalyzed transfer of the phosphoryl-choline head group to Cer (Figures 2d and e), unlike the cellular PI pool of control and *smpd3−/−* chondrocytes, which contained 18:0/20:4-DAG core as major species (Supplementary Figure S3),^30^ the well-known activator of protein kinases (PKC)^31^ in the reversible recruitment of DAG-responsive proteins with C1 subdomains to PLM.^30, 31, 32^ Potential intricate regulatory functions of Cer and DAG in the Golgi complex await further investigations.

SM/C enriched DIMS of GC membranes are the scaffold of SMPD3 and SMS1 proteins (Figures 1i and k). Sophsticated atomic force microscopy and fluorescence correlation spectroscopy have been applied to phase-separated lipid bilayer model systems, consisting of ternary SM/PC/C domains, embedded in a liquid disordered PC phase, to investigate the effect of Cer on liquid-ordered membrane domains.^33,34–34^ Treatment with bacterial nSMase released Cer from SM of the SM/C complex and simultaneously displaced cholesterol stoichiometrically at the rim of ordered nanoscale domain structures. It is tempting to correlate results obtained from these model systems with those of this study on the role of SMPD3 in liquid-ordered DIMS of the Golgi membrane complex.

Complementary and in support of our results are the observations that protein trafficking to the cell surface is retarded in response to downregulation of SMS1,^36^ and the Golgi secretory function inhibited at reduced DAG levels.^30^

SMPD3 induced changes in the homeostasis of SM/PC/Cer/DAG concentrations in the lipid pool of the GC are expected to be minor and quantification impeded by insufficient current methodology. Kinetic studies of Cer and DAG release, SM hydrolysis and phosphoryl-choline group transfer from PC in control and SMPD3-deficient GC membrane stacks may give insight into the molecular mechanism underlying the potential role of SMPD3 in the Golgi SMPD3/SMS1 cycle in remodeling the Golgi lipid bilayer membrane for vesicle formation, transport and secretion.

Collectively, these studies suggest a novel mechanism underlying the function of SMPD3 in the GSP and necessitate future experiments addressing the concerted action of SMPD3, SMS1, and of Cer and DAG lipid-driven remodeling of the membrane bilayer in the GSP.

This unprecedented aspect of SMPD3 function necessitates a reconsideration of the current tenet regarding the role of Smases and SM metabolites in intracellular signal transduction pathways. Expansion of this concept to other rapidly growing cells, for example, in tumors, the immune system and inflammation, awaits further studies and may open new perspectives for therapeutic strategies.

## Materials and Methods

### Mouse experiments

All experiments were approved by the Institutional Animal Care and Research Advisory Committee of the University of Cologne, Cologne, Germany.

Generation and genetic characterization of *smpd3−/−* mice (C57Bl/6 × 129 background) have been described.^13^ They were backcrossed ten times into the C57BL/6 background.

### Egfp-N2 fusion constructs

Full-length *smpd3*cDNA,^2^ inserted into the pcDNA 3.1 myc/his vector, was amplified with primers NSM2 5′*Xho*I-sense (5′-CTCGAGATGGTTTTGTACACGACCCCCTTTCTT-3′) and NSM2 3′*Kpn*I as (5′-GGTACCACGCCTCCTCTTCCCCTGCAGACACCA-3′) and ligated into the *Xho*I-*Kpn*I restricted pEGFP-N2 vector.

Full-length *smpd4* RNA was amplified by RT-PCR using primers smpd4s*Xho*I 5′-CCCTCGAGGTCTGCTATGGCGTTCCCTCAC-3′ and smpd4 as *Bam*HI 5′-ATGGCCAGCAGCTCGGATCCGGTGCAGCTT-3′. Smpd4 cDNA was ligated into the PCR2.1 Vector (Invitrogen, Karlsruhe, Germany), isolated as *Bam*HI-*Xho*I fragment and ligated into the pEGFP-N2 vector.

### Transfection

C57Bl/6 embryonic fibroblasts (EMFIs) and HEK293 cells were grown in Dulbecco's modified Eagle's medium supplemented with 10% horse serum, and transfected by electroporation with expression vectors *smpd3—EGFP*-N2 and *smpd4-EGFP*-N2.

### Real-time PCR

RNA was isolated for RT-PCR from p16 control and *smpd3−/−* chondrocytes using Trizol (Invitrogen), reverse-transcribed using a Transcriptase kit (Life Technologies Inc., Darmstadt, Germany).^37^ Quantitative PCR reactions were performed with the ABI Prism 7900HT using a 96-well format, the Fast SYBR Green Master Mix (Applied Biosystems, Waltham, MA, USA), following the manufacturer's protocol. Data analysis was performed using the 2-ΔΔCt method.

### Acid and neutral sphingomyelinase assays

Total aSMase- and Mg^2+^-dependent nSMase activity were determined as described.^38^

### SM synthase assay

SMS1 activity in cell lysates was determined following an established procedure.^23^

### Cell fractionation

Cell fractionation and isolation of Golgi fractions and Triton X-100 insoluble DIMs were performed by established procedures^39,40–41^

### Cytokine analysis

Cytokines, secreted by peritoneal macrophages via the non-Golgi-secretory pathway, were quantitated in the medium using the BioPlexPro mouse cytokine 23-plex assay (Bio-Rad, Bioplex #M600009RDPD, Hercules, CA, USA) following the manufacturer's protocol. Macrophages were isolated from adult (4mo) male control and *smpd3−/−* mice 72 h after intraperitoneal injection of 1.5 ml 4% thioglycolate, washed with PBS. In all, 1.5 × 10^6^ macrophages unstimulated and stimulated with 100 *μ*g LPS/ml medium for 48 h were used. The supernatant was centrifuged for the cytokine assay.

### Cell culture

EMFIs (3–6 passage) derived from e14 C57bl/6, C57bl/6 × 129 *smpd3−/−* (ref.13) and HEK293 cells were grown in Dulbecco's modified Eagle's medium (Sigma, Taufkirchen, Germany), supplemented with 10% fetal calf serum (Life Technologies Inc.), 2 mmol glutamine, 100 units/ml penicillin and 100 *μ*g/ml streptomycin in a humidified incubator at 37 °C in a 5% CO_2_ atmosphere. Primary chondrocyte from p16 control and *smpd3−/−* of femur epiphyseal cartilage and rib cages were cultured following established procedures.^22^

### Lipidomic analysis

Isolation, separation, identification and quantification of phospho classes and SL classes were analyzed by MS/MS using an Applied Biosystems QTrap analyzer (Darmstadt, Germany).^41^ In brief, total lipids were isolated following the method of Bligh and Dyer.^42^ Phospho and SLs were separated by HPTLC in solvent system chloroform/ethanol/triethylamine/water 60/70/70/14 (v/v/v/v). Bands were identified by charring with 10% CuSO_4_.5 H_2_O, 8% H_3_PO_4_ at 180 °C for 5 min and quantified using the CAMAG Scanner and software (CAMAG, Muttenz, Switzerland).^43^

Lipid bands were visualized with primuline for analysis by MS/MS using an Applied Biosystems QTrap analyzer.^41^

### Protein analysis by western blotting

Protein aliquots were analyzed by WB, using the following antibodies: anti-SMS1, anti-Igf1R and anti-Igf (Santa Cruz Biotechnology, Heidelberg, Germany), anti-MSH (Dianova, Hamburg, Germany), anti-Col2a (Millipore, Darmstadt, Germany), anti-COMP, anti-ColVI, anti-ColIX (kindly provided by M Paulsson, Center for Biochemistry, University of Cologne, Cologne, Germany), anti-Arf and anti-Annexin V-Cy3 Apoptosis Detection Kit (Alexis Biochemicals, San Diego, CA, USA), anti-GHRH (Inserm, Paris, France), anti-pIRE and anti-ATF6 (Abcam, Cambridge, UK), anti-Caveolin (BD Transduction Laboratories, Heidelberg, Germany), anti-K58 and anti-*β-*Cop (Sigma, Taufkirchen, Germany), affinity purified anti-SMPD2 and SMPD3 (Ser310 – Ala655) polyclonal antibodies,^1^ mouse anti-matrilin 3 (Matn3) antibodies (kindly provided by R. Wagener,^44^ Center for Biochemistry, Cologne, Germany). Quantification was carried out by densitometry using the IMAGE J2X program (Rawak Software, Informer Tech Inc).

### Immunofluorescence microscopy

Primary chondrocytes of p10 control and homozygous female *smpd3−/−* mice were grown to semiconfluency on cover slips or on Aclar membranes, respectively, fixed for processing for light and immunofluorescence microscopy with 4% paraformaldehyde in PBS and permeabilized with PBS/0.5% Triton X-100, 4 °C. After blocking with 3% BSA/PBS, cells were treated with respective antibodies in TBS supplemented with 5% non-fat dry milk at 4 °C over-night. After washing with PBS/0.5% Triton X-100, cells were incubated with Cy3-conjugated second IgG antibody (Jackson Immuno Research, Baltimore, PA, USA) for 1 h at 37 °C, washed with PBS/0.5% Triton X-100, and analyzed by epifluorescence using a Zeiss Axioplan Imager M1 (Oberkochen, Germany) or Leica confocal microscope (Wetzlar, Germany).

### FACS analysis of single-cell chondrocytes

For cell survival experiments, cultured primary chondrocytes were analyzed by flow cytometry as previously described.^45, 46^ Briefly, chondrocytes were isolated by collagenase type 2 treatment (Worthington Biochemical Corporation, Lakewood, NJ, USA) and subsequently stained with fluorescently labeled annexin A5 (AnxA5-Dye490)^2^ and Sytox Blue Dead Cell Stain (Thermo Fisher Scientific, Schwerte, Germany) in binding buffer (10 mM HEPES, 140 mM NaCl, 2.5 mM CaCl_2_, pH 7.4) followed by flow cytometry analysis (FACSCantoII, Becton Dickinson, Heidelberg, Germany). Using the FlowJo 7.6 software (LLC Ashland, OR, USA), cell debris were excluded and by comparing the area and width of the FSC and SSC signals only single cells were considered for further analysis. Viable (AnxA5^-^/Sytox Blue^−^), apoptotic (AnxA5^+^/Sytox Blue^−^) and dead cells (AnxA5^+^/Sytox Blue^+^) were quantified and data represented as mean±S.D. of *n*=4 individual mice with three technical replicates each. Statistical analysis was performed with Student's *t*-test using the unpaired two tails method.

### Electron microscopy

Primary chondrocyte were grown on Aclar-membranes (Fa. TED Pella Inc., Redding, CA, USA) for 6 days, washed 3x with PBS, fixed for 1 h at 4 °C with 2% glutaraldehyde, 2% PFA, 0.2% picric acid in 0.1 M cacodylate buffer, pH 7.35. Fixation buffer was removed and cells washed 3x with 0.1 M cacodylate buffer, pH 7.35 post-fixed in 1% OsO_4_ solution for 1 h, and stained in 1% uranyl acetate for 1 h at room temperature. After dehydration, specimens were embedded in Araldite (Serva, Heidelberg, Germany). Ultra-thin sections (about 70 nm) were stained with uranyl acetate and lead citrate and were examined by EM (Zeiss 902A, Zeiss, Oberkochem, Germany). The semi-thin sections (1 *μ*m) were stained with methylene blue for light microscopy.

### Statistical analyses

Results are expressed as mean±S.D. Statistical significance of differences between two groups was determined by a two-tailed Student's *t*-test using GraphPad QuickCalcs (La Jolla, CA, USA): *t*-test calculator. A *P*-value of *⩽0.05, **⩽0.01, ***⩽0.001 was considered significant. Sizes of animal cohorts are listed under respective figures.

## Supplementary Material

Supplementary Inforamation
