# Supplementary material for: Neutral sphingomyelinase (SMPD3) deficiency disrupts the Golgi secretory pathway and causes growth inhibition
Source: Cell Death Dis. 2016 Nov 24;7(11):e2488–. doi: 10.1038/cddis.2016.385 (PMC5260872; doi:10.1038/cddis.2016.385)
Supplement: Supplementary Inforamation [file cddis2016385x1.pdf]

Neutral sphingomyelinase (SMPD3) deficiency disrupts the Golgi secretory pathway and causes growth inhibition

Wilhelm Stoffel*[,1, 2], Ina Hammels[1, 2], Britta Jenke[1], Erika Binczek[2], Inga Schmidt-Soltau[1], Susanne Brodesser[2], Astrid Schauss [2], Julia Etich[3], Juliane Heilig[3], Frank Zaucke[3,4]

1. Center of Molecular Medicine (CMMC), Laboratory of Molecular Neurosciences, Center for Biochemistry, Faculty of Medicine, University of Cologne, Cologne, Germany
2. Cluster of Excellence, Cellular Stress Response in Aging-Related Diseases (CECAD), University of Cologne, Cologne, Germany
3. Center for Biochemistry, Faculty of Medicine, University of Cologne, Cologne, Germany
4. Dr. Rolf M. Schwiete Research Unit for Osteoarthritis, Orthopedic University Hospital, Friedrichsheim gGmbh, D-60528 Frankfurt/Main, Germany

*Corresponding author. Tel. +49 221 478 6881; Fax: +49 221 478 6882: E-mail: wilhelm.stoffel@uni-koeln.de

SMPD2, SMPD3 and SMPD5 are members of a superfamily of Mg^{2+}- dependent phosphohydrolases, clustered in the neighbor-joining dendrogram [1]. The DNA sequence of SMPD4 [2], however, shared neither sequence homology nor active site motifs of the catalytic domain matched by the superfamily profile. We studied the role of SMPD4 in HEK293 cells, stably transfected with a *smpd4-egfp* fusion construct and confirmed the subcellular topography of SMPD4-EGFP predominantly in the GC [2]. However, sensitive nSMase assays [3] in cell lysates of these cell lines revealed no increase of nSMase activity beyond that of *wt* HEK-cells, **Supplementary Figure S1b**. Our experiments preclude the bona fide SMPD4 as an nSMase and pointed to SMPD3 as the dominant mammalian nSMase.

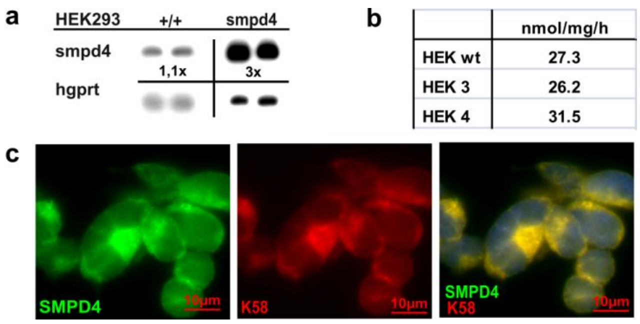

Supplementary Figure 1. Enzymatic activity and immunohistochemistry of stably expressing *smpd4-egfp* HEK cells preclude SMPD4 as a neutral sphingomyelinase. (a) qRT-PCR of cRNA of wt and *smpd4-egfp* transfected HEK293 cells, (b) neutral sphingomyelinase activity was not increased in overexpressing *smpd4-egfp* transfected HEK cells, clones HEK 3 and 4. (c) Fluorescence images of *smpd4-egfp* overexpressing HEK cells indicated SMPD4-EGFP localization in the Golgi complex (green). Merged images indicated colocalization with Golgi marker K58 (red) in the Golgi complex.

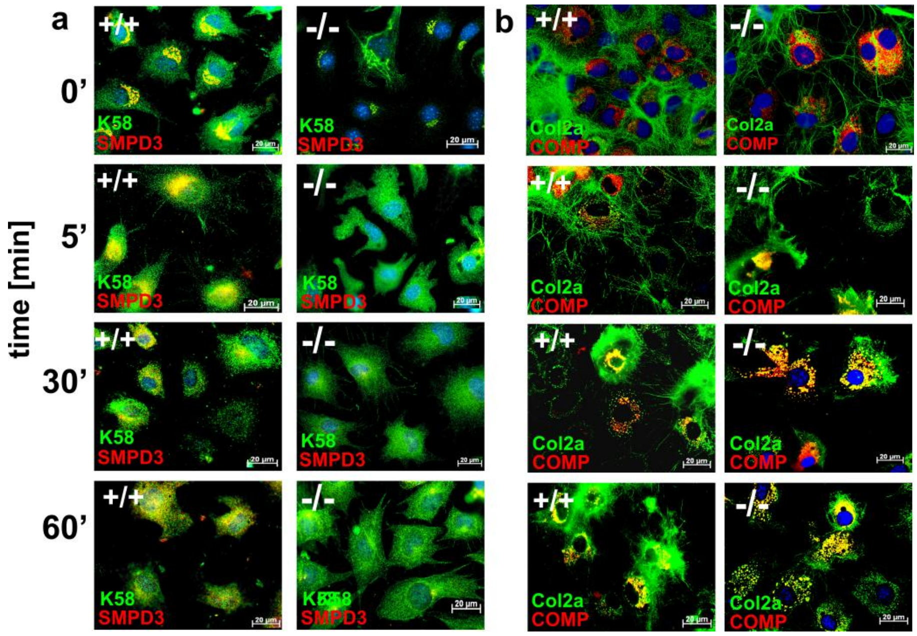

Supplementary Figure 2. Time resolved disintegration of Golgi complex in wt and *smpd3-/-* chondrocytes on exposure to brefeldin. **(**a) Fluorescence images of p10 wt and *smpd3-/-* primary chondrocytes, grown in culture for six days on cover slips treated with 5µg BFA/ml for 5, 30 and 60 minutes and double stained with: SMPD3- (red) and K58-antibodies (green), **(**b) Col2a- (green) and COMP-antibodies (red).

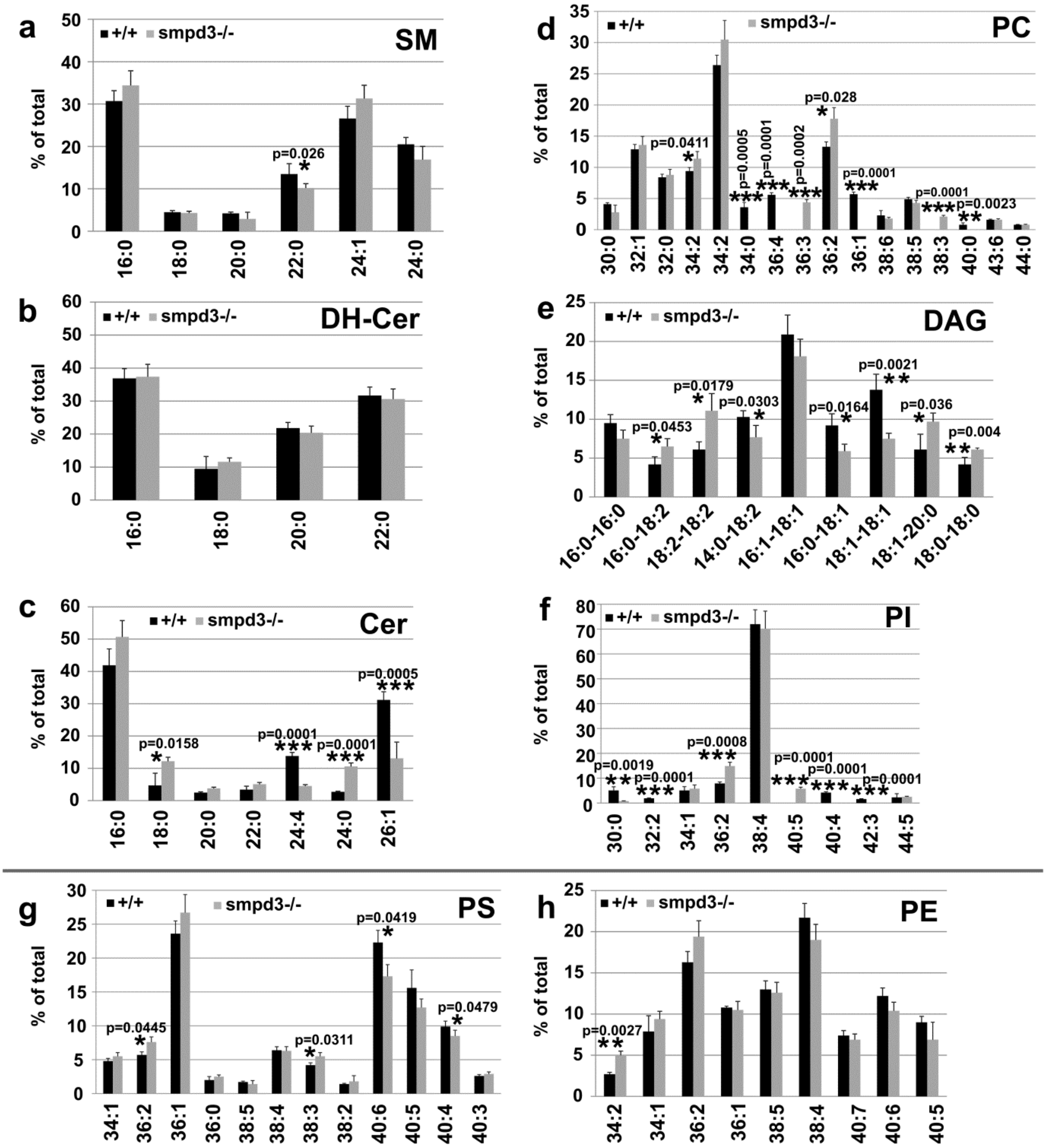

Supplementary Figure 3. Phospholipidome of wt and *smpd3-/-* chondrocytes**. (**a-h) HPTLC-separation of SPM, Cer and PL classes of total lipid extract of wt and *smpd3-/-* chondrocytes. Quantitative MS/MS-analysis of sphingomyelin (SM) (a), dihydroceramide (DHCer) (b), ceramide (Cer) (c), phosphatidylcholine (PC) (d), diacylglycerol (DAG) (e), phosphatidylserine (PS) (f), phosphatidylinositol (PI) (g) and phosphatidylethanolamine (PE) (h). N=3

Supplementary table1.

Primers for real time-PCR

smpd1 s	5'-cctttatcaaccttaaccctggctaccgag-3'	elov4 as	5'-ctgatggcgtaggcga-3'
smpd1 as	5'-cgaagcttttgcctcaggtagataagcagc-3	elov5 s	5'-gtatggctgggaccaa-3'
smpd2 s	5'-ctctgaaaaccactacaggctgtgaccctc-3	elov5 as	5'-catatcggattctcccgc-3'
smpd2 as	5'-ctgggtctccgtttcccttgtcagaacgtg-3'	elov6 s	5'-acccgaactaggtgac-3'
smpd3 s	5'-ctggctgatttccgaaaatctacctcctcg-3'	elov6 as	5'-cgggagactcggaaac-3'
smpd3 as	5'-gtcaggccggacagctgggtgataaaactg-3'	elov7 s	5'-gggaacattccatgcc-3'
sms1 s	5'-atgctaacgctcacctacctatttatcaa-3'	elov7 as	5'-gcaacctctgaccctt-3'
sms1 as	5'-tagtcggcagagctgttatgtgtcgtttac-3'	bmp1 s	5'-gtgtgggggccaagtgcgggcagatgtgaa-3'
sms2 s	5'-acttctggtggtatcacttggtctgctggc-3'	bmp1 as	5'-tcacttcctgctgtggagtgtgtcctggaa-3'
sms2 as	5'-gctcaggtagacttctcattatcctccccg-3'	bmp2 s	5'-caagccaaacacaaacagcggaagcgcctc-3'
degs s	5'-gcttttagacccctgttc-3'	bmp2 as	5'-tgctaacgacacccgcagccctccacaacc-3'
degs as	5'-gtagtgcgggaggtca-3'	bmp4 s	5'-cacctcatcacacgactactggacaccaga-3'
kinase s	5'-tcatgtccggtgatgg-3'	bmp4 as	5'-tttaccgtgagtcaagtcacccctgtgttg-3'
kinase as	5'-ggcttgctaggcgaaa-3'	comp s	5'-gagcagacgtactggc-3'
lyase s	5'-atgtctgctaaggggt-3'	comp as	5'-gggagaagcagaagaca-3'
lyase as	5'-cggggtccgtagtataa-3'	Col2a s	5'-ccacttcagctatggc-3'
spt s	5'-tatgccacgtcgatgt-3'	Col2a as	5'-cggtactcgatgacgg-3'
spt as	5'-actcaatgatcggggt-3'	sox9 s	5'-ctacccgcccatcacccgctcgcaatacga-3'
kdsr s	5'-gacaagctactgcaggcgaagaaagacatt-3'	sox9 as	5'-ctgtgtgtagactggttgttcccagtgctg-3'
kdsr as	5'-cacagtgacgtacacattgtacggcttcac-3'	cerS1 s	5'-agtgggcacttgtcgtacccgacggttgca-3'
a cer s	5'-gcatgaattattggctcaaaaggcaccagc-3'	cerS1 as	5'-caacggcagccacactcatccaccaccatg-3'
a cer as	5'-gaatcctgtcaacatgcccacatatccaac-3'	cerS2 s	5'-acgcgggatggaagaacacctgcaacaacc-3'
igf1 s	5'-cacctcttctacctggcgctctgcttgctc-3'	cerS2 as	5'-ttagctaggagccggctctttgctcctgcc-3'
igf1 as	5'-tctgagtcttgggcatgtcagtgtggcgct-3'	cerS3 s	5'-ccctgttcttcatcttcaccgtcgtcttct-3'
elov1 s	5'-ccaaagctaccctctg-3'	cerS3 as	5'-ctaacggccatgctgaccattggcaatgag-3'
elov1 as	5'-actcgaaccatccgaag-3'	cerS4 s	5'-cacgtctctgtgatgactccatcaagaact-3'
elov2 s	5'-ctcctactacggcctg-3'	cerS4 as	5'-gcgggccattggtaactgctactctaggcc-3'
elov2 as	5'-ccgtcatgccattagc-3'	cerS5 s	5'-ttggcgcagcttttatagttttcgtctagc-3'
elov3 s	5'-gacttcgagacgtttcag-3'	cerS5 as	5'-gcagttggcaccattgctagagctgctgcc-3'
elov3 as	5'-cacggtttgcttgagg-3'	sec23 s	5'-agcacagtggcggaagtcaggataccaggac-3'
elov4 s	5'-ggattggaatcaagtggg-3'	sec23 as	5'-tgcaagtttcttcaaatgatccatgaacac-3'

References

1 Hofmann, K., Tomiuk, S., Wolff, G. & Stoffel, W. Cloning and characterization of the mammalian brain-specific, Mg2+-dependent neutral sphingomyelinase. *Proc Natl Acad Sci U S A* **97**, 5895-5900, (2000).

2 Krut, O., Wiegmann, K., Kashkar, H., Yazdanpanah, B. & Kronke, M. Novel tumor necrosis factor-responsive mammalian neutral sphingomyelinase-3 is a C-tail-anchored protein. *The Journal of biological chemistry* **281**, 13784-13793, (2006).

3 Tomiuk, S., Hofmann, K., Nix, M., Zumbansen, M. & Stoffel, W. Cloned mammalian neutral sphingomyelinase: functions in sphingolipid signaling? *Proc Natl Acad Sci U S A* **95**, 3638-3643, (1998).
